# Editorial

**Published:** 1855-10

**Authors:** 


### Editorial.

### American Convention of Dentists.

We had not the pleasure of attending this convention which was held on the first Wednesday of August last, in Philadelphia; and we regret very much that circumstances prevented us from the rich treat we then promised ourselves.

Our experience fully convinces us of the Professional importance of such convocations. We hold that no man can be so high as not to be benefitted by such intercourse, we are all learners, and however much we may differ as to the best mode of organizing and conducting such convocations, yet, with the proper spirit carried out, much individual good must be the result.

We regret to see that some of our Brethren have misunderstood the objects of the meeting, and that it has been the cause of any invidious feeling. The character of the Profession is somewhat in keeping by every member, and all should feel an interest in its promotion.

We might as well condemn any means for the dessemination of knowledge, all our periodical publications, &c., &c., as to condemn public discussions on professional subjects; in the latter way may we alone expect to be benefitted by the experience of many who never write, and who are yet very proficient operators.

So far as we understand it, the "American Convention" is not an exclusive association, neither is the object the settlement of constitutional law or professional etiquette, but the discussion of *practice.* We love a courteous professional Brother and if he is a *true* gentleman, he is sure to be such. We also love to know the particular mode of performing every operation in Dental Surgery, and hence we have taken great pleasure in reading the report of the discussions held during the sitting of the convention, and which we give our readers in the present No. of the Register,—that those who could not be present may understand what they have missed, and with us try and be at the next meeting.

With our present feelings we should like to see State and Local associations, with delegates to a national society. This would then be what its name imports ; yet until the former becomes more general, the latter is to some extent impracticable.

### Gutta Percha for the Base of Artificial Teeth.

We feel assured the Profession will be glad to learn that this material has at last been brought to that condition which will warrant the supposition that it may be made available in Mechanical Dentistry.

Since writing the note to the article by Dr. Slayton, which will be found in the present No. of the Register, we have seen an upper dentine made of the material, also seen an upper set which has been worn for several months, and cannot see any reason why it may not answer all for which it is claimed.

The whole operation is very simple, and indeed differs but little from what has already been described as the mode of putting up such work. The material however appears harder, requiring a greater anount of heat to work it, but the coloring is certainly much more perfect than we expected at present to see. We are now experimenting and fully expect by compression in metallic dies, to make the base *plate* far more compact and firm, also less bulky in the mouth. If this succeeds, as we believe it will, we shall feel free to use it in many partial operations, and apply it to all temporary operations for full or half sets when even a small portion of gum can be advantageously used in the operation. The following note from Dr. Hunter is published by request of Dr. Slayton :—

Cincinnati, October 3rd, 1855,

Dr. Slayton,—Dear Sir,—

In answer to your enquiry concerning your Gutta Percha work, I answer that I think it susceptible of *great* good in mechanical Dentistry in such cases as are selected with judgment.

For temporary work it *must* be of utility, or at all events such is my conviction, and it is my determination to test it.

Yours respectfully, Wm. M. HUNTER.

### Ohio College of Dental Surgery.

The regular course of Lectures commences in this Institution the first Monday in November, the prospects for a good class are good. Students have commenced coming in to avail themselves of the infirmary course which is open during this month. We have been several times asked, will Allen's Continuous Gum work be taught in this Institution? We answer that it will. The Professor of Mechanical Dentistry teaches all the different modes of inserting teeth, will give practical illustration in every method, so that students may understandingly avail themselves of every improvement in the Profession.

### Dental Register.

We now issue the first Number of Vol. IX. It will be seen that the work is materially enlarged and is now furnished to subsrribers at $3.00 per annum. It is hoped that as far as possible payment will be made in advance, and to encourage this we now purpose to take Five Dollars for vols. nine and ten if paid by the issuing of the January number.

### OBITUARY.

We have the painfnl duty to announce the decease of Dr. Wm. B. Ross, of Newport, Ky., who came to his death by a very distressing casuality on the 5th inst.

Dr. Ross was crossing the street near his dwelling about 3 P. M., when a horse attached to a milk wagon came running down the street and in attempting to stop the horse by catching the reins, it is supposed fell and the shaft of the wagon struck him on the temple, entering the brain, and killing him instantly. We know of no man who has filled so many different spheres of life, with so general and universal acceptance—as a minister of the Methodist Episcopal Church, he was efficient and zealous, and continued his duties as such until a disease of the throat compelled him to desist—as a Dental Practitioner, he maintained an enviable reputation, and on the organization of the Ohio College of Dental Surgery he entered the first session and graduated with honor—having been in practice previous for several years. For the last three or four years he has been out of practice, but still maintained his interest in the profession.

At his decease he was acting magistrate of Newport, and member of the Town Council. In all the avocations of life as minister, Dentist, Magistrate, Councilman, etc. he had the confidence of the community. He leaves an interesting family and numerous friends to mourn his loss.

---

THE REPORT OF THE TRIAL OF DR. JOHN ALLEN *vs.* DR WM. M. HUNTER.

This Report gotten up in an extra of the Dental Register, is still for sale at the several places where first advertised, and will be sold hereafter at 50 cents. Those having them for sale, will please observe this notice.

